# Developing an experimental model of early knee osteoarthritis after medial meniscus posterior root release: an in vivo study

**DOI:** 10.1186/s40634-022-00501-y

**Published:** 2022-07-09

**Authors:** Lika Dzidzishvili, Irene Isabel López-Torres, Carlos Carnero Guerrero, Emilio Calvo

**Affiliations:** 1 Department of Orthopaedic Surgery and Traumatology, Hospital Universitario Fundación Jiménez Díaz, IIS-Fundación Jiménez Díaz, Universidad Autónoma de Madrid, Av. De los Reyes Católicos, 2, 28040 Madrid, Spain; 2 Animal Core Facility Manager, Experimental Surgery Department and Animal Core Facility, IIS- Fundación Jiménez Díaz, Universidad Autónoma de Madrid, Av. De los Reyes Católicos, 2, 28040 Madrid, Spain

**Keywords:** Knee osteoarthritis, Meniscal root tear, Animal model, Early osteoarthritis, Rabbit model

## Abstract

**Purpose:**

To develop a predictable and reproducible model of knee osteoarthritis after medial meniscus posterior root release.

**Methods:**

Posteromedial meniscal root tears were created in 12 White New Zealand rabbit knees. The contralateral limbs were used as healthy controls. The animals were euthanized at 16 weeks postoperatively; tissue samples of femoral and tibial articular cartilage were collected and processed for macro and microscopic analyses to detect signs of early degeneration. Clinical evaluation of the weight-bearing status on the affected knee was conducted at 0-, 4-, 8-, and 16-weeks postoperatively.

**Results:**

Early and severe osteoarthritic changes were the hallmark and the main findings after 16-weeks post-surgery. Macroscopically, extensive osteoarthritic changes were observed across the femoral condyle and tibial plateau. Microscopic finding included ulcerations, fissures, fibrillations, pitting, and loss of the superficial layer. Cellularity was diminished, the normal pattern of distribution in columns was lost, and subchondral bone exposure was also evident.

**Conclusions:**

This study describes a novel model of knee osteoarthritis that may guide the development of tailored interventions to delay or prevent knee osteoarthritis. This knowledge could shift the current treatment paradigm toward more conservative and knee salvageable treatment options and increase surgeons’ awareness of this injury pattern. Such considerations may have a positive impact on clinical decision-making and subsequent patient-reported clinical outcomes.

**Design:**

Controlled laboratory study.

**Level of evidence:**

II.

## Introduction

Knee osteoarthritis (OA) is a leading cause of pain and disability in the elderly and a rapidly expanding public health concern, both in terms of health-related quality of life and financial expenditure.

Over the last decades meniscal root tears has gained increased attention and been considered as a silent pandemic [1]. Medial meniscus posterior root tears (MMPRT) have been strongly associated with osteoarthritic changes in the knee and are considered biomechanically equivalent to a subtotal meniscectomy [2].

By nature, osteoarthritis studies in humans have limited potential to describe early onset of degenerative changes and to predictably evaluate disease progression. Moreover, OA of the knee is frequently diagnosed as an end-stage disease requiring joint replacement surgery. Cadaveric studies have also been widely employed to investigate meniscal root tears [3, 4]; however, models based on cadavers are static and fail to replicate the natural history of OA. These limitations may be overcome by developing experimental animal models.

Despite the wide use of animal models for knee OA research, to the best of our knowledge no experimental model for knee OA after medial meniscus posterior root release has been developed. In this context, the question that arises is whether it is possible to develop a reproducible model of knee OA after MMPR release. We hypothesized that surgical destabilization of a MMPR in an experimental rabbit model could predictably induce knee OA.

For this reason, the purpose of the present study was to develop a predictable and reproducible model of knee OA and to describe early radiological and histological changes after MMPR release.

## Material and methods

The research complies with national legislation and was approved by the animal protection service and institutional review boards of the authors' affiliated institutions as well as the regional ethics committee (PROEX 297.8/21).

### Experimental animal model

A total of 12 adult New Zealand male rabbits weighing approximately 2,5–3 kg were used. Animals were housed in individual cages that allow easy access to standardized food and water ad libitum and were given 1 week to acclimate to the housing facility. During housing, animals’ health status was monitored daily.

The animals were anesthetized with an intramuscular injection of 50 mg/kg of ketamine (Ketolar®, Parke-Davis, Barcelona, Spain) supplemented with 10 mg/kg of xylazine (Rompun®, Bayer, Leverkusen, Germany), and received metamizole as an analgesic (20-40 mg/kg), intramuscular injection during the first postoperative day.

**Fig. 1:**
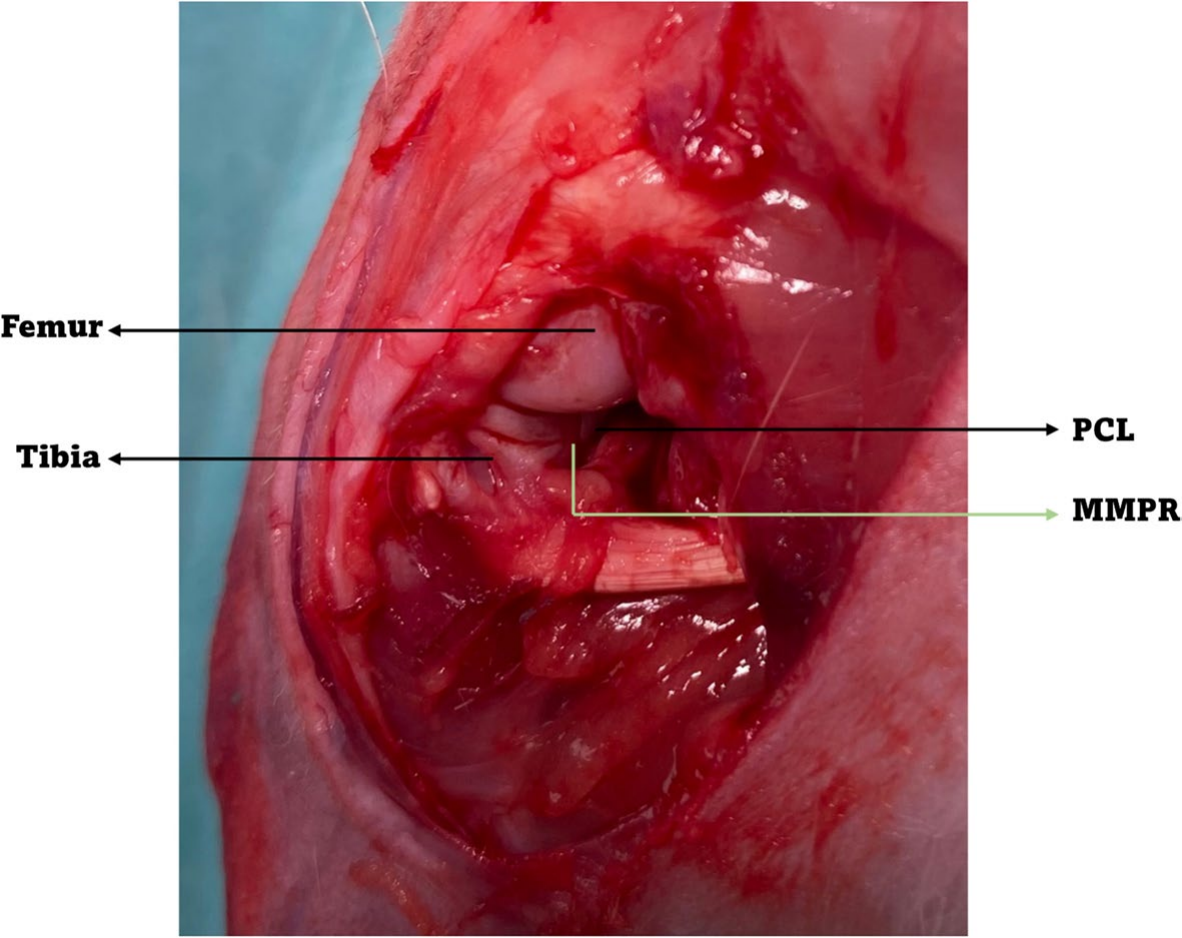
Meniscectomy was performed by transecting the medial meniscus posterior root with a radial cut (PCL, posterior cruciate ligament; MMPR, medial meniscus posterior root)

All surgeries were performed by the same orthopedic surgeons at the same facility and partial meniscectomy was conducted after medial meniscus posterior root transection to experimentally induce knee OA. The posterior-medial aspect of each hindlimb was shaved and the knee was approached through a 3-cm incision over the posteromedial aspect under sterile conditions. The approach was extended through the subcutaneous tissue medial to the superficial branch of the saphenous vein. The posterior aspect of the joint capsule was identified and the arthrotomy was created between the medial head of the gastrocnemius and medial collateral ligament (Fig. 1).

The medial meniscus posterior root was completely transected from its tibial insertion with a number 11 surgical blade. After sectioning with a scalpel, the meniscal roots were probed to verify that they had been completely detached from their root attachments. Iris scissors were used to remove the posterior horn of the meniscus. The collateral and cruciate ligaments were not damaged by this approach. The arthrotomy was closed in a layer fashion with a continuous No 2–0 Vicryl® (Ethicon, Somerville, NJ, USA) suture and skin closure was completed with a continuous Dafilon*®* (Nylon, B. Braun, Rubi, Spain) monofilament suture. A soft dressing was applied for 1 week following surgery. All animals were permitted free cage activity after surgery, were monitored postoperatively, and returned to normal activity in individual cages. Clinical evaluation of the weight-bearing status on the affected knee was conducted at 0-, 4-, 8-, and 16-weeks postoperatively. An independent observer rated the weight-bearing of the animal by awarding one point for no weight-bearing, two points for partial weight-bearing and three points for full weight-bearing.

The rabbits were euthanized by administration of intracardiac sodium pentobarbital (50 mg/kg) (Pentotal, Abbott, Madrid, Spain) at 16-weeks postoperatively and the knees were harvested. The follow-up was set at 16-weeks as we considered this would be a proper timing to examine development of osteoarthritic changes in the rabbit knee and based on previously published studies using time points of 2-, 6-, and 12- weeks [5–7].

### Histopathological analysis

**Fig. 2:**
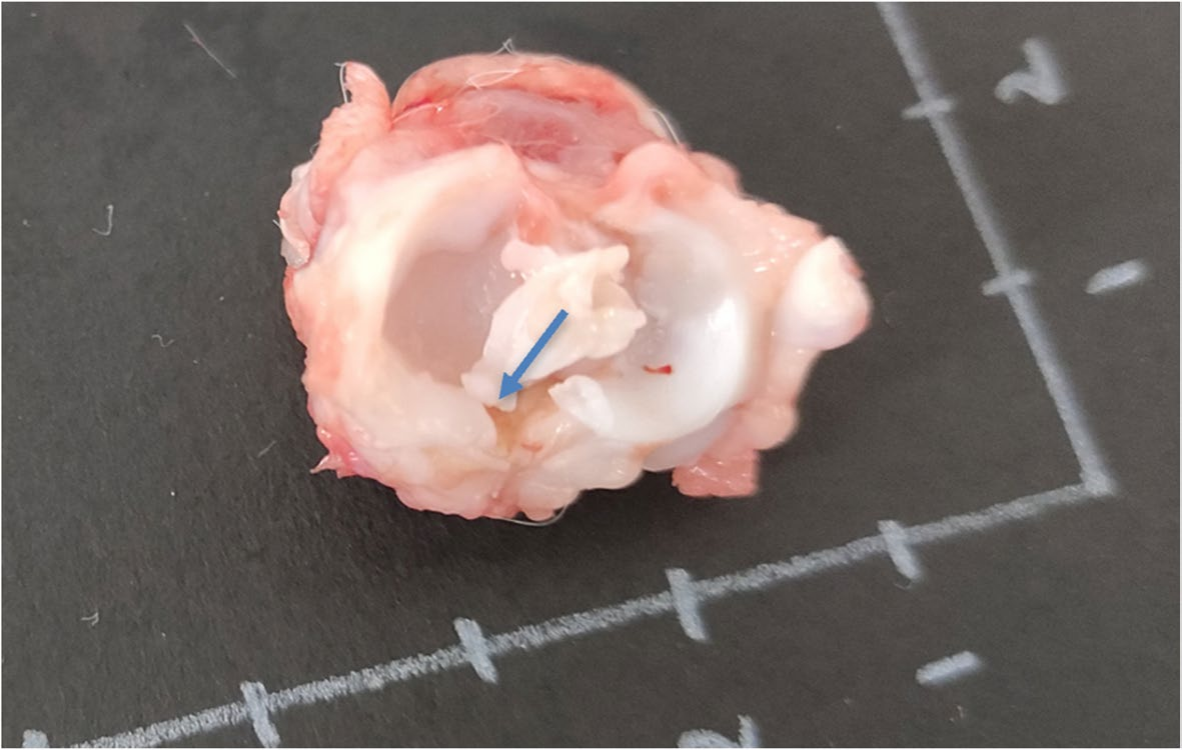
Representative image of meniscal root remnant after 16-weeks of partial meniscectomy

All the knees were carefully dissected, and the infrapatellar synovial pad excised in order to evaluate macroscopically. The two femoral condyles, the tibial plateau, and the menisci were isolated for macroscopic evaluation (Fig. 2). Images were acquired using a 10_objective lens under bright field and then digitally overlaid. Contralateral knees were managed following the same protocol and used as healthy normal controls.

For the femoral condyle and tibial plateau cartilage, the severity of macroscopic changes was categorized as 0 (normal smooth surface), 1 (surface rough with minimal fibrillation or pitting), 2 (cartilage erosions extending into the superficial or middle layers), 3 (full-thickness erosion or osteophytes), 4 (complete cartilage erosion with subchondral bone exposed).

For the microscopic examination, isolated femoral condyles and tibia plateaus were fixed in 10% buffered formalin for 24 h and then decalcified for 48 h with formic acid combined with formalin for further histological evaluation. Formic acid was used since it is the weakest descaling agent, thus avoiding degradation of the sample, which, combined with formalin, allows us to carry out fixation simultaneously.

**Fig. 3:**
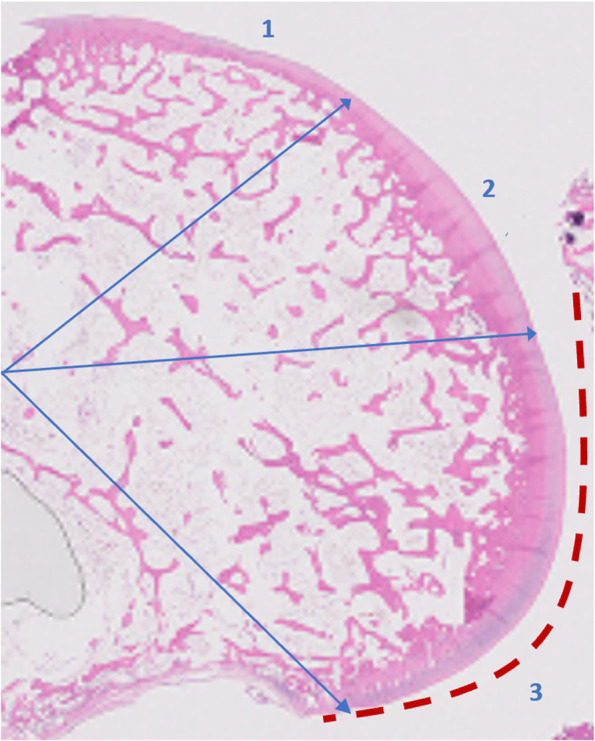
Sagittal section of medial femoral condyles was divided in three zone of interest (1. anterior, 2. central, and 3. posterior). The red dotted line shows the weight-bearing area of the medial femoral condyle

The decalcified knee joints were cut in the sagittal plane along the mediolateral central portion of the articular surface of each medial femoral condyle corresponding to the weight-bearing area, as previously described [8]. Sections (2 μm) were embedded in paraffin and stained with hematoxylin–eosin to assess cellularity and structural abnormalities, and with alcian blue to evaluate matrix abnormalities. For histopathological analysis, medial femoral condyles were divided in three equal regions of interest (anterior, central, and posterior) Fig. 3. Region-of-interest corresponding to the weight-bearing area (zone 2 and 3) of the femoral condyle as rabbits have a characteristic locomotion pattern, they walk on a flexed hind leg. This region was delimited as it shows the earliest and most severe histological abnormalities [8].

Each sample was histopathologically assessed by an experienced cartilage pathologist using the AORSI grading and staging system [9]. A partial score for the categories of the AORSI scale was allocated as grade was defined as OA depth progression into cartilage and each stage was defined as the horizontal extent of cartilage involvement. The final score was defined as the sum of grade and stage representing a combined assessment of OA severity and extension [9]. Moreover, the weight-bearing area (zone 2,3) was compared to non-weight bearing area (zone 1) and the average score of each medial femoral condyle was compared with the mean score of the articular surface of each medial tibial plateau.

### Clinical evaluation

Clinical assessment of the weight-bearing status on the affected knee was conducted at 0-, 4-, 8-, and 16-weeks postoperatively. An independent observer rated the weight-bearing of the animal by awarding one point for no weight-bearing, two points for partial weight-bearing, and three points for full weight-bearing.

## Results

One rabbit undergoing experimental meniscal root release and subsequent meniscectomy has died following surgery. Consequently, data from 11 rabbits were available.

**Fig. 4:**
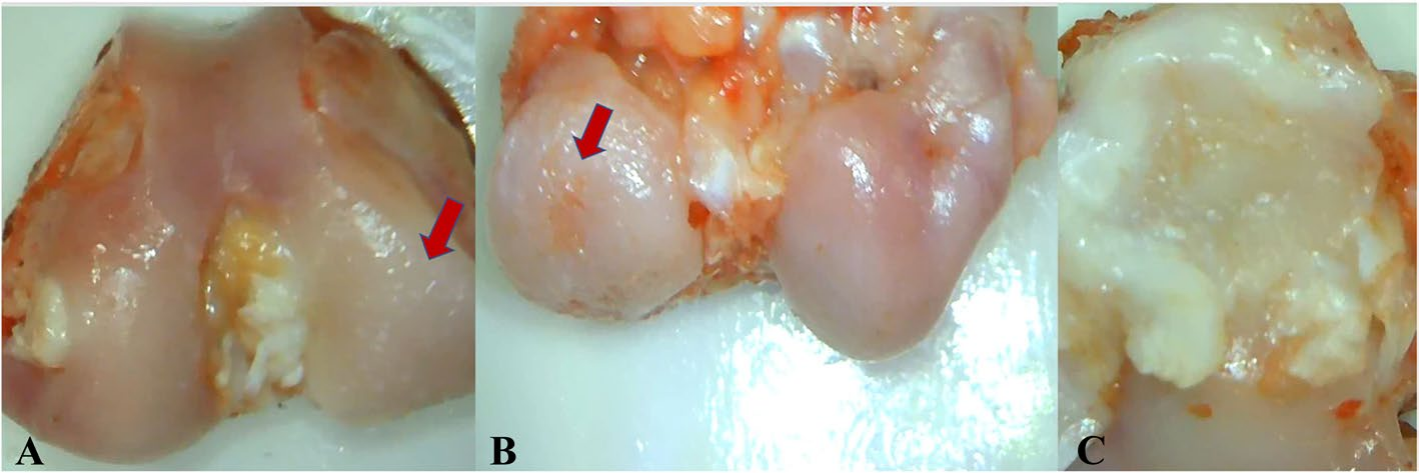
Gross pathological macrographs after 16-weeks from surgery where the load bearing area of femoral condyles appears eburnated, irregularities and pitting can be detected (red arrow, 4**A**). Note full-thickness ulcer in the weight bearing area of the medial femoral condyle with irregularities and pitting (red arrow, 4**B**). The rim of the medial tibial plateau shows a whitish osteophytes and erosions (4**C**). Images were acquired using a 10_objective lens under bright field. Images were then digitally overlaid

Macroscopically, extensive osteoarthritic changes were observed across the medial femoral condyle and tibial plateau especially. The load bearing area of femoral condyles appeared eburnated with full-thickness ulcers predominantly localized at the medial and posterior aspects of the femoral condyle, irregularities and pitting were also evident (Fig. 4A). Although they were distributed along the whole articular surface, cartilage lesions were more intense in the weight bearing area of both femoral condyle and tibial plateau (Fig. 4 B, C). The rim of the medial tibial plateau showed whitish osteophytes bordering the medial compartment of the tibia (Fig. 4C). The synovium showed signs of severe inflammation with hyperplasia in comparison with control knees.

**Fig. 5:**
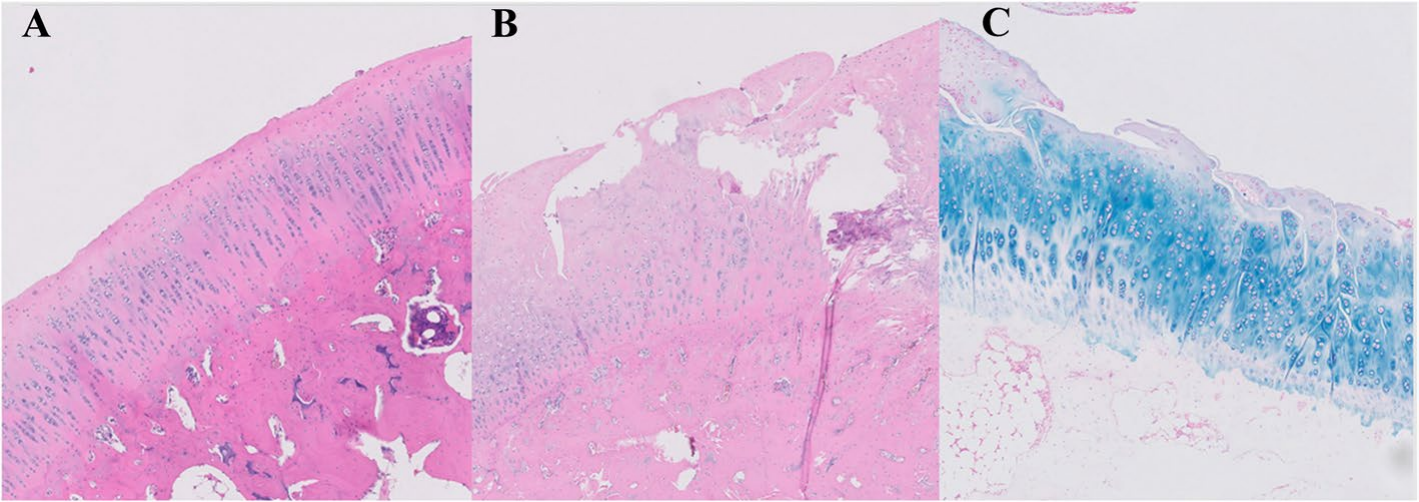
Hematoxylin eosin (5**A**, 5**B**) and alcian blue staining (5**C**) of articular cartilage samples. The cartilage shows a normal appearance, the surface is smooth, chondrocytes are present throughout, and the matrix staining is homogeneous in the control knee (5**A**). The superficial and intermediate layers of cartilage are lost, and matrix vertical fissures reach the deep radial zone in this sample corresponding to an ulcer at the weight bearing area from an OA knee in animals subjected to meniscal root injury and partial meniscectomy (5 **B**, **C**). Cellularity is diminished and the normal pattern of distribution in columns is lost. Cartilage matrix is loss with delamination and excavation of superficial and mid layer (5**B**, **C**). Marked structural abnormalities and hypocellularity are also evident. Matrix staining intensity is severely reduced (× 20)

**Fig. 6:**
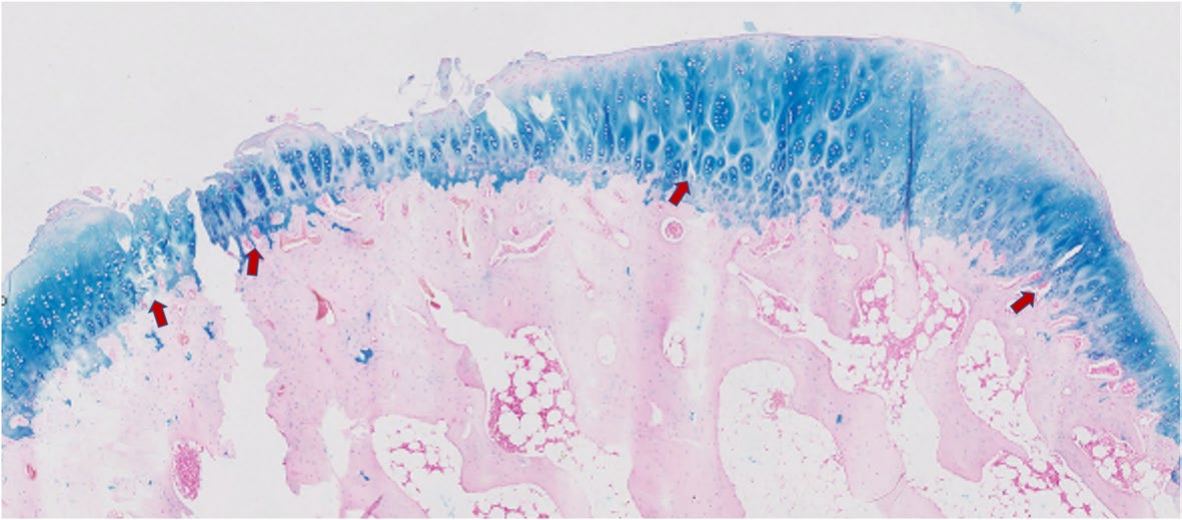
Alcian blue staining of weight bearing articular cartilage sample showing reduction of matrix staining intensity and breached tidemark by blood vessels and with minor splits (red arrows) compromising the integrity of the junction between the calcified and the noncalcified cartilage

Microscopically, structural irregularities consisted of fibrillation, ulcerations and matrix vertical fissures reaching the deep radial zone. Specimens showed a mild to moderate reduction of the matrix staining with delamination and excavation of superficial and mid layer fissures. Regarding cellular changes, knees revealed mild alterations in chondrocytes, cellularity was diminished, the normal pattern of distribution in columns was lost, and infiltration of subchondral bone were also evident (Fig. 5). Cartilage was usually hypocellular, and clones could be appreciated in the superficial and deep zones of the tissue. The chondrocytes were distorted with increases in activated large mononuclear cells and hemosiderin deposits were observed in the specimens as it has previously been related to degenerative joint disease [10]. Surface structural irregularities consisting of focal loosening of the lamina splendens and mild fibrillation could be observed early in most of the knees where the medial meniscus posterior root had been transected. The tidemark disruption was found in most sections breached by blood vessels and minor splits compromising the integrity of the junction between the calcified and the noncalcified cartilage. (Fig. 6).

Partial and total scores corresponding to the grading and staging categories of the AORSI classification and comparison between femoral non-weight bearing (zone 1) and the weight bearing area (zone 2, 3) were conducted, as well as between the femoral and tibial articular surfaces.

Interestingly, microscopic cartilage surface changes showed a trend to be more severe in the weight-bearing area of the medial femur than in the non-weight bearing area and tibia. The combined histological scores were statistically significant between the different zones (Table 1).

**Table 1:** AORSI scores, and comparison between femoral non-weight bearing and the weight bearing area, femur, and tibial articular surfaces

AORSI	Medial femur (non-weight bearing area)^a^	Medial femur (weight bearing area)^a^	Medial Tibia^a^	*p*-value ^ non-weight bearing vs weight bearing	*p*-value ^ Femur vs Tibia
Grade	2 (1)	4 (1)	2 (0.5)	< 0.001	0.002
Stage	1 (0)	4 (1)	2 (1)	< 0.001	0.006
Total score	2 (0.5)	16 (7)	6 (3)	< 0.001	< 0.001

^a^Values are represented by median and interquartile range

^ Significance level of *p* < 0.05

**Fig. 7:**
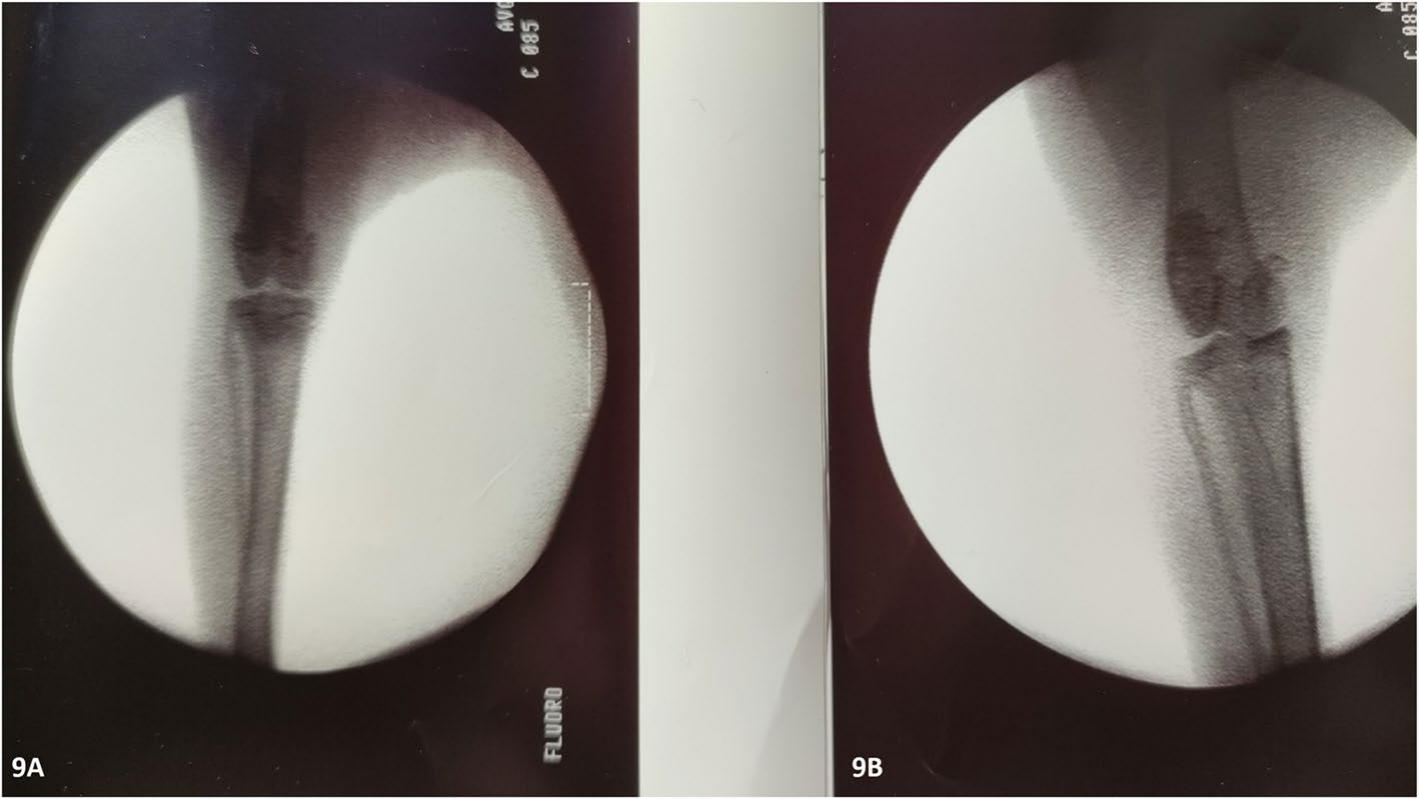
Comparative radiographic images just before (9**A**) and after 16-weeks post-surgery (9**B**) showing medial joint space narrowing

Conventional radiography continues to be the primary imaging technique used to assess the severity and progression of OA. Degenerative changes and joint space narrowing were evident comparing pre- and 16 weeks post- surgery plain Rx (Fig. 7).

Regarding postoperative clinical outcomes, meniscal surgeries temporarily caused moderate unloading of the knee of the operated limb, however, shortly all animals had mostly recovered presenting no pathological walking patterns. Subjective assessment revealed gait disturbance patterns beyond the 8-weeks post-surgery as the animals showed partial weight bearing status.

## Discussion

The current study described a novel, in vivo model of knee OA after medial meniscus posterior root transection. Early and severe osteoarthritic changes were the hallmark and the main finding of this experimental model after 16 weeks post-surgery in rabbits subjected to partial meniscectomy after meniscal root injury. Macroscopically, extensive osteoarthritic changes were observed across the femoral condyle and tibial plateau. Microscopic changes were marked by the ulcerations, fissures, fibrillations, pitting, and loss of the superficial layer were dominant mainly in the medial compartment and weight-bearing area.

Currently no reliable method exists in humans for OA detection at a reversible stage. Frequently, histological studies are performed with tissue obtained at the time of joint replacement surgery, which represents end-stage disease and where lack of tissue from early/preclinical disease restricts more detailed research. Therefore, it is of a paramount importance to develop animal models of knee OA where early degenerative changes can be induced reliably. Various animal models of osteoarthritis have been developed to study the disease process under more controlled conditions, where stages of progression can be better defined and evaluated. In addition, animal models can detail the time-course of disease and provide information on the pathophysiology and/or molecular mechanisms of the degenerative process.

The choice of a particular animal model involves numerous factors. The ideal animal model should induce consistent and reproducible disease in a suitable time frame. Ideally, the animals should be mammalian species that are easy to house, inexpensive, and allow different outcome measurements (i.e., radiological, histological, and clinical). The model should enable assessment of clinical outcomes such as gait abnormalities. There are; however, limited clinical outcomes currently available in many of the species used. To our best knowledge, there is the only one sheep model of OA in which gait abnormalities have been evaluated [11]. Our model is the first to describe gait abnormalities after meniscal injury in rabbits.

Animal size is another important consideration. Small animals are advantageous in terms of costs, housing, and genetic manipulation. Whereas large animals are most advantageous in terms of anatomical and biomechanical similarities to humans, and the major simplicity to use diagnostic imaging. Rabbits are one of the most commonly used animals as experimental models of OA for being medium-sized animals, easy to handle, and are phylogenetically closer to humans than most species. Experimental models in rabbits after meniscectomy procedure can induce reproducible disease and can also have a counterpart in human beings, where knee OA is directly related to partial meniscectomy [7]. Colombo et al., [7] reported outcomes after lateral meniscectomy in the knees of rabbits. Significant degeneration was observed with ulceration, osteophyte formation, fibrillation, and loss of chondrocytes in the articular cartilage. Lesions were documented as early as 1–2 weeks post-surgery and increased in number and severity up to 12 weeks.

One of the most widely used experimental models of OA is the anterior cruciate ligament transection (ACLT) model and numerous studies have also been published [12–15]. Common histological findings in the articular cartilage of the ACLT model, similar to ours, include loss of matrix staining, fissures, cartilage erosions, chondrocyte loss and clustering. Tidemark integrity however is preserved in the ACLT models [15]. Our findings can be interesting as meniscal root injury not only increase the prevalence of early degenerative changes, but also the severity of the cartilage lesions correlated with the advanced stage of OA and verifies a direct relationship between this particular type of meniscal tear and knee OA. In our model the ACL was left intact in this way we prevented a potential confounding effect of ACL tear on knee OA. Moreover, unlike lateral meniscus posterior root tears that are mainly traumatic and frequently associated with ACL injury, medial meniscus posterior root tears are usually degenerative meniscal tears.

It has been demonstrated that the decrease in matrix staining, followed by changes in cellularity are the earliest histological abnormalities detected in OA, while breaches of the tidemark and injury to cartilage structure are only prominent at advanced stages [8]. Our findings suggest that medial meniscal root injury can induce advanced degenerative changes and almost selectively affects the medial compartment accelerating cartilage damage in the weight-bearing area.

Regarding histological examination, standard scoring systems are not universally available. The most commonly used histopathology grading system is the one described by Mankin [16], though the relationship between the pathological parameters scored and clinically relevant disease is not well established that makes it difficult to compare results between studies. The present study addressed this difficulty by using AORSI classification system [9]. This classification system has made as a consensus which favors a better uniformity of the controlled means of study and the results reported in research on animal models of osteoarthritis.

The current body of literature suggests an emerging paradigm in injury-inflicted osteoarthritis. A novel experimental model described here induced reproducible high-grade disease after a suitable time frame following meniscal root injury. The degenerative changes caused in this model are thus confirmed, both microscopically and macroscopically, being comparable with those observed in humans in a more accessible, highly reproducible way. These changes can be a promising therapeutic target, which promotes the interest of this model of osteoarthritis induced by medial meniscal root injury. Investigation of early osteoarthritic changes may lead to understanding of when and how rapidly knee OA develops after meniscal root injury. This would shift the current treatment paradigm toward more conservative and knee salvageable treatment options.

There are limitations of this model that should be considered when reviewing. The control knees used in this study were nonoperative controls. The influence of inflammation produced on these results is unknown. However, we discussed in detail and preferred to use healthy knees to avoid introducing bias due to joint inflammation. Additionally, using the contralateral knees as controls may have skewed results due to altered weightbearing; however, animal gait returned to normal a couple of days after surgery, so the impact on the results was rather negligible.

The main purpose of this study was to determine whether the medial meniscus posterior root injury will predictably produce measurable knee OA. Since the present model was able to develop early OA model, future investigation reporting outcomes between different therapeutic options for the medial meniscus posterior root tears is warranted.

## Conclusions

This study describes a novel model of knee osteoarthritis that may guide the development of tailored interventions to delay or prevent knee osteoarthritis. This knowledge could shift the current treatment paradigm toward more conservative and knee salvageable treatment options and increase surgeons’ awareness of this injury pattern. Such considerations may have a positive impact on clinical decision-making and subsequent patient-reported clinical outcomes.

## Abbreviations

OA: Osteoarthritis
MMPRT: Medial meniscus posterior root tear
MRI: Magnetic resonance imaging
AORSI: Osteoarthritis Research Society International
ACLT: Anterior cruciate ligament transection
